# Role of Residues Undergoing Hereditary Spastic Paraplegias Mutations: Insights from Simulating the Spiral to Ring Transition in Katanin

**DOI:** 10.1021/acs.jcim.5c00421

**Published:** 2025-04-21

**Authors:** Maria S. Kelly, Riccardo Capelli, Ruxandra I. Dima, Paolo Carloni

**Affiliations:** † Department of Chemistry, University of Cincinnati, Cincinnati, Ohio 45221, United States; ‡ Department of Biosciences, Università degli Studi di Milano, 20133 Milano, Italy; § INM-9, Forschungszentrum Jülich, 52428 Jülich, Germany

## Abstract

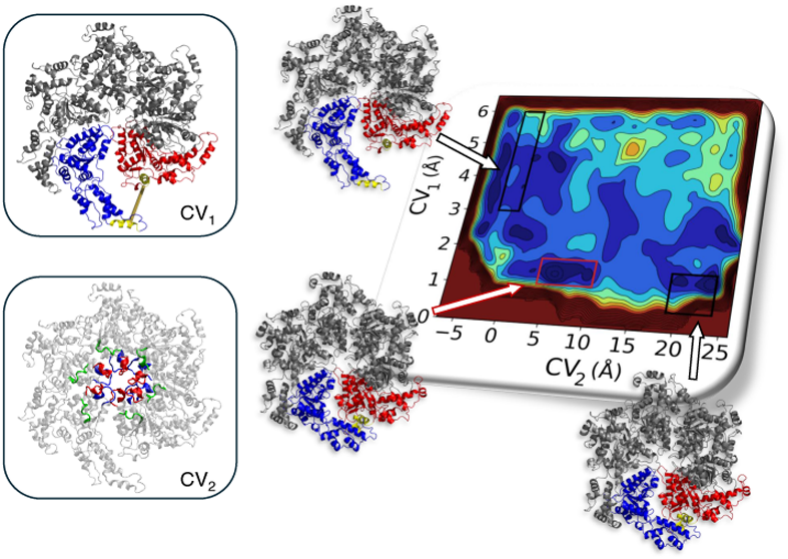

Several dozen mutations in the M87 isoform of the spastin enzyme have been associated with mobility impairment in hereditary spastic paraplegias. Some of them impact the structural determinants of two functional conformations of the protein: spiral and ring. Here we investigate the possible patterns between these disease-related residues in spastin and aligned regions in the closely related protein katanin toward their role in the transition of the two conformations, which is essential for both enzymes’ function. By performing a variety of molecular simulations (including metadynamics) on katanin, we suggest that about one-fourth of the known M87 spastin disease-associated mutations also affect the interconversion and/or the stability of a previously unrecognized intermediate of the katanin transition. The protocol used here can be applied to the study of conformational changes in other large biomolecular complexes.

## Introduction

The most predominant isoform of the human spastin protein (M87, corresponding to the UniProt entry Q9UBP0-3 and missing the first 86 residues compared to the canonical spastin entry) is a microtubule severing enzyme that is essential for shaping the microtubule network within neurons, particularly in axon growth and maintenance.^1−3^ The protein (hereafter S-M87) removes tubulin dimers from microtubules in order to modify their lattices.^4^^,^^1^

S-M87 dysfunction is associated with neurodegenerative disorders that can lead to partial or complete loss of mobility of humans, named hereditary spastic paraplegias.^5−9^ As many as 65 missense mutations in S-M87 have been associated with the disease (Table S1).^10^ Some of them affect S-M87’s ability to bind ligands and oligomerize into its functional quaternary structure (Table S1).^10^

**Figure 1:**
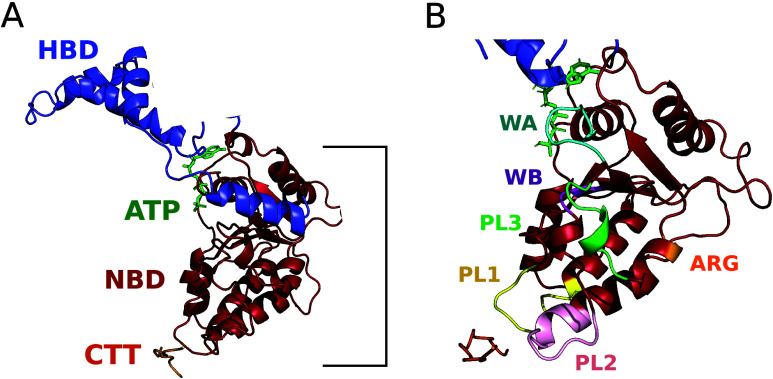
(A) Katanin monomer with domains (NBD and HBD) and ligands (ATP and CTT) labeled in their respective colors. The details of the binding site, with the Mg(II) are shown in Figure S1. (B) Katanin’s NBD domain with functional motifs colored and labeled. C-terminal helix in the HBD was removed for visual aid.

**Figure 2:**
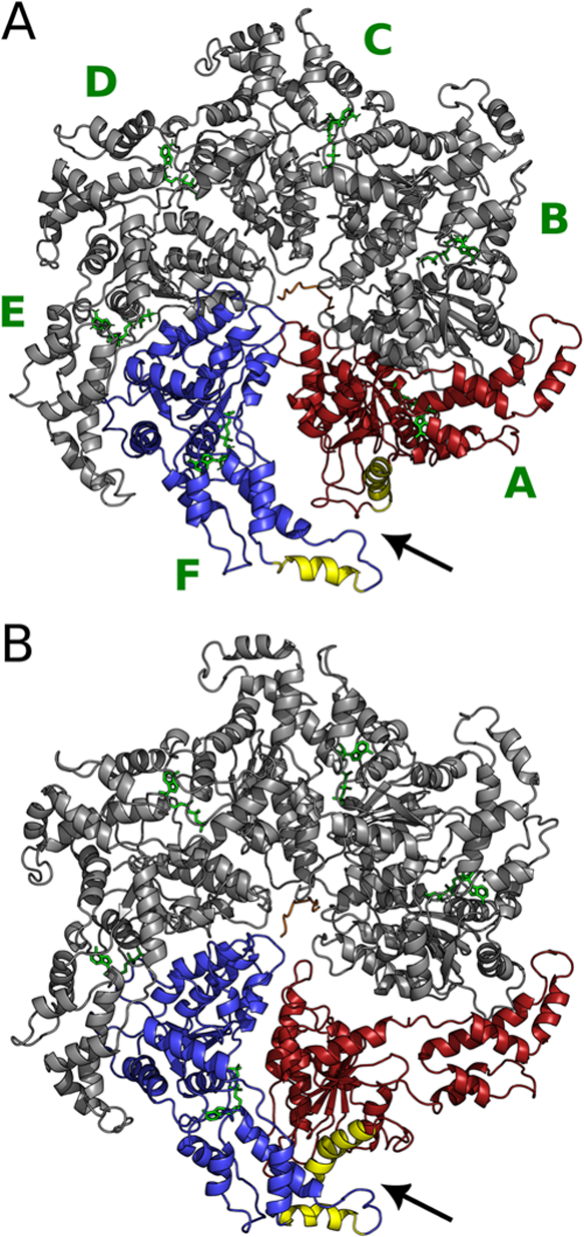
Katanin spiral (A) and ring (B) conformers. The protomers A-F are labeled. The terminal protomers A and F are colored red and blue, respectively. The two α helices used for CV_1_ are colored in yellow. Protomers A-F are labeled in the spiral. The HBD–NBD interaction and the entrance of the CTT into the hexamer’s pore are shown with an arrow. The MIT domains were unresolved in the cryo-EM structures and, therefore, are not depicted here.

S-M87 belongs to the ATPases Associated with diverse cellular Activities (AAA+) superfamily of proteins. It shares function and most of its structural determinants with the AAA+ protein katanin:^11−14^ (i) The *nucleotide-binding domain* (NBD), responsible for ATP binding and hydrolysis (“ATPase motor”). As shown by cryoelectron microscopy (cryo-EM, Figure 1), the ATP-binding pocket is formed by the *Walker A, Walker B*, and *Arg-finger* motifs. The cofactor is stabilized by a Mg(II) ion. The NBD interacts with the C-terminal tails of tubulin (CTTs). These are directly exposed to the mechanical forces generated by the enzymes, leading to the severing of the microtubules.^2,12^ Two positively charged pore loops (PL1 and PL2), located in the NBD and protruding into the central pore, form a spiral around the negatively charged CTT of the microtubules (required for substrate processing; Figure 1). Residues in the pore loop 3 (PL3) form hydrogen bonds with both the nucleotide and PL2, thus connecting ATP and CTT binding (Figure 1).^2^ (ii) The *microtubule-interacting and trafficking* (MIT) domain, formed by a three-helix bundle. This provides anchoring support for the ATPase motor to make contact with the microtubules: a flexible linker region that connects the MIT with the NDB,^11,15^ enabling proper orientation of the latter on the microtubule lattice (Figure 1). (iii) The *Helical Bundle Domain* (HBD), unique for these two proteins across the AAA+ family (Figure 1). The HBD of each protomer binds to the NBD of the neighboring protomer, forming an asymmetric spiral hexamer with a large gate between the two terminal protomers (Figure 2).^12,13,16^ This movement allows the entrance of the CTT into the hexamer’s pore where the PLs are located (Figure 2). ATP hydrolysis in one of the two terminal NDBs (Figure 2) is the driving force of the transition of the spiral structure to the ring structure (S → R, Figure 2), where the loss of the nucleotide within protomer A results in the closure of the terminal gate.

The binding of ATP, CTT, and the formation of the protomer–protomer interactions are thus essential parts of the severing mechanism.^1^ An important question is the following: do katanin and S-M87 share similar functional dependencies on residues that affect the spiral-ring transition? To begin addressing this issue, here we uncover the structural determinants of the S → R transition, so far unknown, using molecular simulations. We focus on katanin from *Caenorhabditis elegans* because of the availability of more structural information on this enzyme than for S-M87, and consider comparing the severing enzymes based on the disease-related mutation locations of S-M87 since we know their functional relevance.

Given the large size of the hexameric complex (consisting of 1910 amino acids), we first investigate qualitatively the S → R interconversion in katanin using an efficient nonequilibrium technique, ratchet&pawl molecular dynamics (MD)^18,19^— similar to steered molecular dynamics^20^—as a function of two apt collective variables (CVs): (i) the distance between two helices of opposite terminal protomers to control the opening and closing of the S → R transition, and (ii) a linear combination of distances between pairs of atoms forming intermolecular interactions (salt bridges and hydrogen bonds) in the ring and spiral states, computed by the dimensionality reduction technique *Harmonic Linear Discriminant Analysis* (HLDA).^21,22^ Next, we use Well-Tempered Metadynamics (WT-MetaD), an accurate technique able to explore the free energy landscape, as a function of the two CVs.^23,24^ We exploit the ability of this technique to trigger the system to escape its free energy minima to find out-of-pathway relevant transients.^25^ Indeed, the simulations successfully identify an intermediate state that rationalizes the mechanistic effects of several disease-related residues, characterizing them as essential for the severing event to take place. This protocol is applicable to large real-world systems, where exhaustive exploration by normal enhanced sampling approaches is out of reach, even with current computational capabilities.

## Materials and Methods

### Katanin System Preparation

The two cryo-EM structures of the katanin complex, namely, the spiral (6UGD, 3.5 Å) and ring (6UGE, 3.6 Å) conformations, were obtained with ATP and a polyglutamate substrate in place of the microtubule tubulin tail.^11,12^ Both structures contained missing residues in the ranges of 183–187 and 324–331 that were modeled using the Modeller program version 9.23.^26^ To model the transition from a spiral to a ring, the nucleotide from protomer A was removed from the spiral to match the configuration of the ring. This aligns with katanin’s conformational transition, where stochastic cycles of ATP hydrolysis and removal from the hexamer result in increased flexibility within the terminal protomer that closes to the ring structure. In both conformations, we also modeled the TUBB sequence of β-tubulin (betaWT) in place of the solved polyglutamate substrate using PyMOL version 3.0 and GROMACS 2022 with the GROMOS 54a7 force field to construct and perform energy minimization of the betaWT sequence, respectively.^27−31^ This sequence was docked in place of the minimal substrate using the GRAMM protein docking server from the Vakser lab by aligning the betaWT sequence to the minimal substrate in order to select the best starting position.^32^

### Molecular Dynamics Simulations

We ran molecular dynamics (MD) simulations of spiral and ring configurations described above using GROMACS 2022.^30,31^ The automated topology builder (ATB) was utilized for ATP parameters.^33^ Each structure (∼19,000 atoms) was placed in a rhombic dodecahedron water box to increase computational efficiency compared to the size of the cubic box, and the water molecules were represented with SPC-16 explicit solvent with 67 sodium ions to neutralize the total charge of the system.^34^ Energy minimization was done with the steepest descent algorithm and the Verlet cutoff scheme^35^ for 50,000 steps. For NVT and NPT equilibration, we used the velocity-rescaling thermostat at 300 K with the leapfrog integrator algorithm and the Parrinello–Rahman barostat at 1.0 bar, respectively.^36,37^ Each equilibration step was run for 500 ps with a thermostat coupling frequency of 0.1 ps and a barostat coupling frequency of 2.0 ps. The short-range nonbonded interactions were calculated using a distance cutoff of 10.0 Å and a dispersion correction for energy and pressure for anything past the cutoff. Particle Mesh Ewald was used for long-range electrostatic interactions.^38^ The LINCS method was used for the bond lengths involving hydrogen atoms, and the simulations were run using a 2 fs integration step.^39^ For each system, we ran for a total of 150 ns and checked the convergence of the trajectories using the backbone RMSD, where the first frame of the production run was used as the reference frame.^40^

### Harmonic Linear Discriminant Analysis

To identify a minimal number of apt collective variables (CVs) which can efficiently represent the spiral-ring transition, we employed a protocol previously successful in capturing complex biological changes within fewer dimensions using harmonic linear discriminant analysis (HLDA).^21,22^

This approach starts with a set of observables (called local descriptors) whose distributions in the states of interest are studied. From the analysis of these distributions, a linear combination of all of these local descriptors is generated that maximizes the separation of the states. For HLDA, the within-class scatter matrix (*S*_w_) used to measure the degree of class separation is calculated using the harmonic average to place more weight on states with smaller variances, where (Σ_A_, Σ_B_) is the multivariate variance for each metastable state.^21^
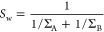
1The collective variable using HLDA (*s*_HLDA_(*R*)) is then calculated in eq 2, with *R* representing atomic coordinates, μ_A,B_ the expectation values per state, and *d*(*R*) the local descriptors.
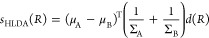
2Here, to gain insight into the interactions that regulate the stabilization of the two states, we collected unique salt bridges and hydrogen bonds between the spiral and ring using unbiased simulations in the ring and spiral states. VMD was used to collect the salt bridges and hydrogen bonds.^41^ Salt bridges were calculated using an oxygen–nitrogen cutoff of 6 Å in attempts to collect more critical differences between the conformations.^25^ For the hydrogen bonds, we set a threshold for the donor–acceptor distance to 3.0 Å with a cutoff angle of 30°. We only considered interprotomer salt bridges and hydrogen bonds except for the two terminal protomers, where we additionally included intraprotomer interactions.

### Ratchet&Pawl Simulations

To test the optimization of our selected CVs and to compare with later metadynamics simulations, we used Ratchet&Pawl MD (rMD) to model katanin’s transition from spiral to ring.^18,19^ rMD is a nonequilibrium technique: after the definition of an apt CV and two states, we can define a transition direction (i.e., we define the starting and the ending states) and we apply a harmonic potential which disfavor the system in going in the opposite direction with respect to the desired one. The harmonic potential term on the CVs helps the system to transition between two states. Such harmonic potential follows the system during the transition, staying still when the system tries to come back to the initial state along the CV. The potential is thus
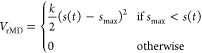
3

where *s*_max_ is the maximum value of the CV during the simulation and *k* is the force constant. Considering this, rMD allows us to study katanin’s transition path between a starting and end state. To describe the hinge-like motion of the terminal protomers in the severing event, we calculated the center of mass distance between an α helix in protomer A (residues 199–215) and protomer F (residues 425–436).^4,10^ These helices were selected based on giving the most extreme distinctions between the spiral and ring state as well as their direct path to forming interactions with one another in the ring. We added another CV using the first eigenvector of the HLDA analysis, using only the top 32 salt bridges or hydrogen bonds that had the largest contribution in distinguishing spiral and ring while also interacting with one of the three functional pore loops to reduce the “noisy” elements that would decrease simulation efficiency while also retaining the connection between katanin’s involvement with the microtubule substrate through the pore loops with the severing motion seen through the spiral to ring transition. We ran 10 simulations of the forward (spiral to ring) and backward (ring to spiral) transitions until katanin had reached the CV boundaries defined in the initial MD runs, which took around 60–70 ns to obtain each transition. The choice of the harmonic constant was made iteratively, starting from an initial value of *k* = 10,000 kJ/mol/nm^2^ and halving it until we observed a swarm of transitions that did not cause partial unfolding in the complex and/or unphysical motions, reaching a value of 500 kJ/mol/nm^2^. Our approach was qualitative and focused on the preparation of the CV. However, it is possible to fine-tune the choice of this parameter by minimizing and differentiating the *k*-values for the two CVs, again using an iterative approach, thus, obtaining a more precise bias potential that can be highly informative in the study of the transition. All CV calculations and rMD were done via GROMACS 2021.6 patched with PLUMED 2.8.^42,43^

### Metadynamics Simulations

We ran WT-Metad simulations using the two CVs from our rMD runs.^23,24^ We performed an initial simulation to survey katanin’s transition from spiral to ring. After 400 ns, we selected 4 configurations from such run to use for a Multiple-Walkers Well-Tempered Metadynamics run.^44^ These include the spiral and obtained ring structures along with two structures found on opposing sides of the obtained landscape to maximize the exploration of conformational space. The bias factor set was 20, and the height of the Gaussians was 1.2 kJ/mol, deposited at a pace of 500 steps. The widths of each Gaussian deposited were 0.05 Å for each of the two CVs. The temperature was set to 300 K. We included upper and lower repulsion potentials using the unbiased MD runs of the spiral and ring states as boundaries for each CV since the calculated HLDA CV is relevant only in describing the spiral-to-ring transition. The multiple-walker simulations continued until the combined total simulation time reached 2.8 μs (700 ns per walker).

### New Intermediate Molecular Dynamics Simulations

To test the stability of the intermediate state identified from the multiple-walker WT-MetaD simulations, we tested it with plain MD simulations. We selected the starting structure for this run by taking the centroid from the *k*-means cluster of the WT-MetaD space that contained only the minimum region. We thus ran 100 ns of plain MD, verifying that the system did not exit the minimum with the same simulation condition detailed in the previous sections.

## Results

We identified two CVs that are able to describe the S → R transition in katanin by monitoring selected distances in 150 ns unbiased MD simulations of both spiral and ring conformers. CV_1_ was defined as the distance between the centers of mass of specific helices from (residues 199–215) and protomer F (residues 425–436) (Figures 2 and 4). CV_2_ incorporates essential changes in the intramolecular interaction network involving the three PLs. As discussed in the Introduction, these are functionally relevant to substrate processing.^11,12^ To distill such information, we aggregate the descriptors linked to the nonbonded interactions (i.e., the distances between pairs of atoms involved in salt bridges and hydrogen bonds) as in ref (25): CV_2_ is then the first eigenvector generated from the HLDA (a linear combination of distances (Table S2)) which best distinguishes the spiral conformer from the ring one. Almost all (97%) of the identified pairs match with corresponding S-M87 residues labeled as disease-related, further confirming the similarity between the two proteins.^4^

**Figure 3:**
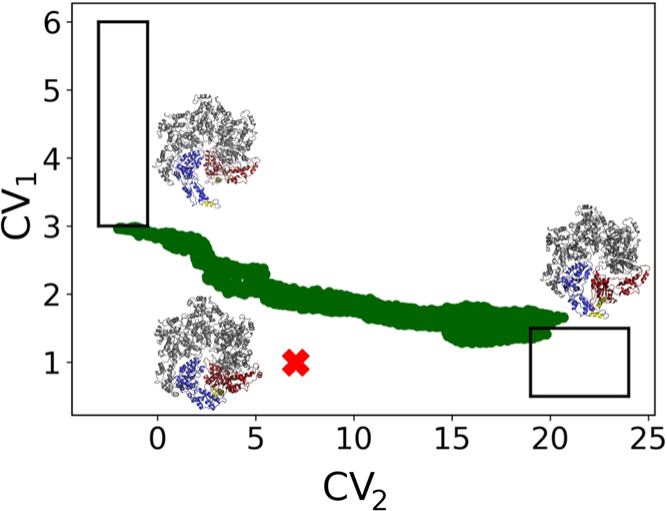
Katanin intermediate as a function of CV_1_ and CV_2_, as predicted by WT-MetaD simulations (red “*X*”). The conformation space predicted by Ratchet-Pawl MD simulations (green region) does not sample the intermediate structure. The boxes represent the spiral (upper left), intermediate (middle), and ring (lower right) structures, colored as in Figure 2. The complete landscape from WT-MetaD is plotted in Figure S2.

Next, we tested the ability of the two CVs to describe the S → R transition by performing CV-based Ratchet&Pawl MD simulations.^18,19^ These simulations led satisfactorily to both forward and backward S → R transitions in a very short time (less than 100 ns), suggesting that the two chosen CVs are appropriate to study this process. The conformational space sampled in this simulation is shown in Figure 3.

Finally, we performed multiple-walker WT-MetaD simulations on four independent replicas for 700 ns each (2.8 μs in total) to investigate the conformational space as a function of the two CVs.

These simulations predicted a complex conformational space associated with the S → R transition (Figure S2). This includes not only the region identified by Ratchet and Pawl MD but also a minimum located outside that region (“*X*” in Figure 3). The Ratchet&Pawl MD was not able to identify this state. To see if the latter really represents an intermediate in the transition, we ran a 100 ns MD simulation starting from a representative conformation of the minimum. We observed that our intermediate structure remained within the region of the minimum for the entire dynamics, confirming the presence of a stable intermediate (Figure 3). The latter is a hexamer with a 40° twist angle, differing from the spiral and ring’s 60° twist (Figure S6). The backbone of the intermediate deviated 5.8 and 6.1 Å compared to the spiral and ring conformer, respectively. Many contacts between the NBDs of protomers A and B present in the spiral conformer and in the intermediate are lost in the ring one (Figure 3 and Table S2). This makes sense since the severing event is associated with a flexibility increase to reach the ring state, which is successfully captured in our simulations.

## Discussion

**Figure 4:**
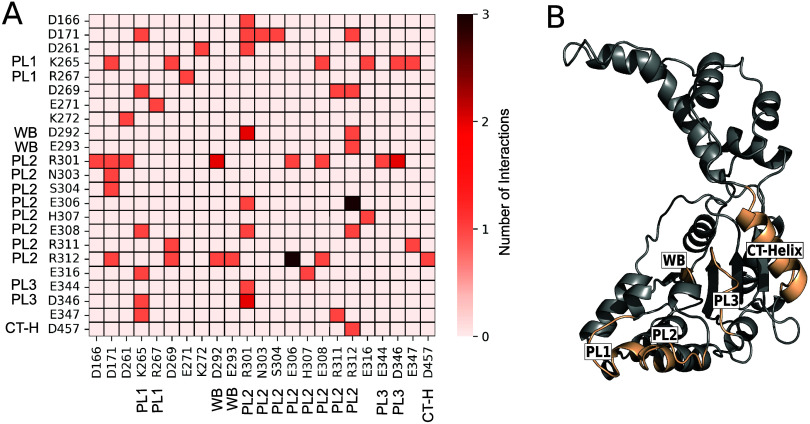
(A) Heatmap displaying the salt bridges/hydrogen bonds included in CV_2_. Residues that make at least one interaction are listed. The number of interactions is indicated by the color bar. Residues that are a part of a functional region are labeled with the latter. (B) Katanin monomer with most functional regions included in CV_2_ labeled.

We have presented a computational study of the S → R transition in katanin, a protein structurally and functionally similar to S-M87, to investigate if katanin’s transition is reliant on specific residues and, in that case, if the corresponding residues in S-M87 undergo hereditary spastic paraplegias-linked mutations. The conformational landscape associated with the transition has been predicted by Ratchet-Pawl MD and metadynamics as a function of two CVs. While the first (CV_1_) is constructed by us (it is simply the distance between selected helices of the protein), the second (CV_2_) is built automatically based on MD simulations of the two conformers. CV_2_ incorporates a variety of geometric features involved in the S → R transition. Strikingly, almost all of the features of CV_2_ (31 out of 32) turn out to involve six residues—conserved on passing from katanin to S-M87 (see Figure 4 and Table S2) and with similar chemical environments (Figure S3A,B)—which undergo disease-linked mutations.^10^ All of them (in boldface hereafter) turn out to play a functional role: **R312** from PL2 (R460 in S-M87) interacts with residues from the CT-helix. This interaction is essential for hexamer stabilization.^10,16,45^**R301** (and the correspondent arginine in S-M87) from PL2 plays a key role in CTT binding, ATP binding, and hexamer oligomerization in both S-M87 and katanin.^10^ It forms salt bridges with **D346** from PL3—important for oligomerization^12^—and with **E293**, a residue in Walker B that coordinates the Mg(II) ion (Figure S4).^10^**D292** is located within the Walker B motif of the NBD. Its disease-linked mutation to a glycine of the corresponding aspartate in S-M87^10^ affects the magnesium binding site and hence ATP hydrolysis. Finally, **R311** in PL2 facilitates interprotomer communication.^12^ The correlation between the disease-related mutations in S-M87 and the corresponding residues in CV_2_ for katanin is fully consistent with the fact that the corresponding residues in S-M87 are crucial to the function of the enzyme.

**Figure 5:**
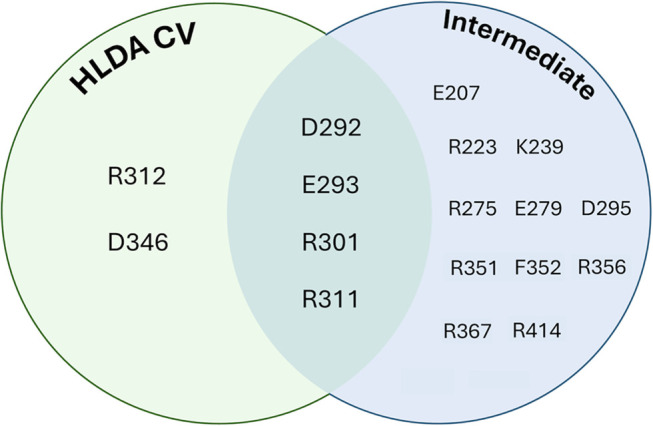
Summary of katanin residues part of CV_2_ and/or forming stabilizing interactions in the intermediate of the S → R transition (Figure 3), fully conserved in S-M87, and undergoing mutations associated with hereditary spastic paraplegias (see Table S1).

WT-MetaD simulations of the S → R transition as a function of the two CVs point to the presence of a previously unrecognized intermediate (Figure 3). Notably, as many as 11 residues, conserved between katanin and S-M87 (Table S2), undergo disease-linked mutations in the latter, forming stabilizing interactions in the intermediate. The chemical environments for all of the residues are similar across the two proteins (data not shown). **K239**, located in the Walker A motif, forms a salt bridge with D292 of Walker B (Figure S5), and it is necessary for ATP binding. The salt bridge made between **E207** and a neighboring protomer’s **R414** stabilizes the hexamer since the latter belongs to the longest α helix on the protomer’s concave interface that makes direct contact with its neighbor. E207 also makes intraprotomer interactions with **R223**, which is necessary for the oligomerization of the hexamer. Here, **D295** in the intermediate interacts with R301 in a neighboring protomer. This points to its role in oligomerization. **R275** is also crucial for katanin’s oligomerization into a functional hexamer.^12^ It forms a salt bridge with E271 that impairs the ATPase activity when mutated. **E279**, in the NBD, forms a salt bridge with R282. The corresponding residue in S-M87 disrupts the folding and stability of the ATPase domain when mutated to a proline.^10^**R367**, located in the HBD, forms an intraprotomer salt bridge with D363. This is essential for the folding of this domain. **R356** in the CT-helix forms a salt bridge with D467. **R351** in the arginine finger is necessary for ATP hydrolysis. It forms an interprotomer salt bridge with D457 to coordinate the arginine finger with the CT-helix of the neighboring protomer. The D292, E293, R301, and R311 disease-related residues also play a role in the stability of the intermediate state. Finally, **F352** in the arginine finger forms a salt bridge with D292, the residue in the Walker B important for Mg(II) ion’s coordination in the neighboring protomer. This interaction makes sense as disease-linked mutations suggest that this residue plays a role in activating ATP hydrolysis in the neighboring protomer. Since the ATP hydrolysis is required for the S → R transition, it is logical that the interactions with this arginine finger residue are present in the intermediate. All of these interactions are present in either the spiral or the ring conformers of the two proteins. Finally, we notice that D292, E293, R301, and R311, which have already been noted, play also a role in the intermediate’s stability (Figure 5).

## Conclusions

Here, we defined a robust protocol that combines the data-driven identification of relevant interactions with enhanced sampling methods to identify structural determinants of conformational transitions in large biomolecular complexes. Applying this protocol to katanin, (i) we ranked the most relevant nonbonded pairs from structural data using molecular simulation-based approaches and (ii) we identified an intermediate along the S → R transition. Because S-M87 and Katanin are two related proteins, sharing the same function (microtubule severing), our findings might allow for a mechanistic interpretation of S-M87 disease-related mutations. Six disease-linked mutations affect the transition, while 11 other positions that undergo mutations in disease are found to stabilize the intermediate (Figure 5). These features might be observed by performing metadynamics simulations of the variants. These features might be observed by performing metadynamics simulations of the variants.

Our approach can be readily adapted to other large protein complexes, enabling the exploration of the system conformational space and the identification of transient states that are essential for function but challenging to capture experimentally.

## Data Availability

All the data and input files needed to reproduce these simulations, as well as trajectory files are available on the public repository Zenodo, which can be retrieved at https://zenodo.org/records/14977029.

## Supporting Information Available

Additional structural information on katanin, locations of CV elements, WT-MetaD results, and a table of S-M87 mutation sites with functions (PDF)

## Footnotes

1 A second isoform, called M1 (the canonical spastin corresponding to the UniProt entry Q9UBP0), is present only in the adult spinal cord. Its involvement in microtubule severing has not been demonstrated. This isoform is not considered in this study.^3^

2 Post-translational modifications of the CTTs (such as glutamylation) regulate the severing activity.^14,17^
