# Supplementary material for: Role of Residues Undergoing Hereditary Spastic Paraplegias Mutations: Insights from Simulating the Spiral to Ring Transition in Katanin
Source: J Chem Inf Model. 2025 Apr 21;65(9):4655–61. doi: 10.1021/acs.jcim.5c00421 (PMC12076492; doi:10.1021/acs.jcim.5c00421)
Supplement: Supplementary file 1 — ci5c00421_si_001.pdf [file ci5c00421_si_001.pdf]

Role of residues undergoing hereditary spastic paraplegias mutations: insights from simulating the spiral to ring transition in katanin

Maria S. Kelly, Riccardo Capelli, Ruxandra I. Dima, and Paolo Carloni

<u>Supplemental Information</u>

Introduction

1. Structural Differences Between Katanin and S-M87

Katanin and S-M87, despite being strongly related, differ in the following structural determinants: (i) katanin has a 'fishhook' motif required for interface interactions between the protomers and S-M87 does not. (ii) katanin has two subunits (catalytic p60 and regulatory p80), while human S-M87 is a homomeric enzyme. (iii) human S-M87 has a dedicated microtubule-binding domain (MTBD), while katanin relies on the p60 subunit for severing and the p80 subunit for regulation. Katanin's p80 subunit and S-M87's MTBD are more important concerning directing and enhancing MT binding.

The ATPase motors between the severing enzymes contain the same functional elements and are composed of an NBD and HBD (*see main text*). However, the cryo-EM structures of their spiral states (katanin, *C.elegans* and S-M87, *D. melanogaster*) show differences in sequence length and secondary structure. There is a 1.13 Å deviation between the backbone of each protein's protomer A. Katanin has 316 residues per monomer while S-M87 has 303 residues (**Chart S1)**. Both domains are slightly smaller in S-M87, with 25 and 24 secondary structures in the NBD and 11 and 9 secondary structures in the HBD for katanin and S-M87, respectively. A larger structural difference is at the HBD tip (denoted by * in the figure below), where S-M87's HBD tip shows a short helix while katanin has a longer flexible loop going into a helix. A previous studies from some of us found this region in katanin to be significantly more flexible than in S-M87, which could influence the stability in the hexamer due to the HBD's role in forming protomer-protomer interfaces.[1]

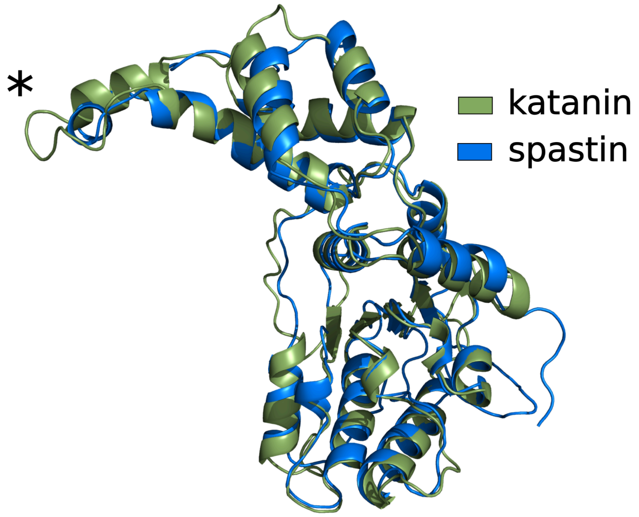

Chart S1

2. Katanin and S-M87: a comparison among structural determinants and function.

We carry out the calculations on katanin from *C. elegans* instead of human S-M87, for several reasons: **(i)** both the spiral and the ring conformations are available only for katanin[3]. **(ii)** The differences between the two proteins are expected not to impact the spiral-ring transition because the different region's functional role is to direct and enhance MT binding.[4,5] **(iii)** The fold, the functional regions, and the vast majority (78%) of the residues undergoing disease-linked mutations are fully conserved across the two proteins (Tab. S2), [6] with very similar chemical environments (as shown by visual inspection) (data not shown).. **(iv)** katanin can partially compensate for the disease-related loss of S-M87 activity in neurons, suggesting a very similar functional role *in vivo* of the two proteins.[7]

3. Figures

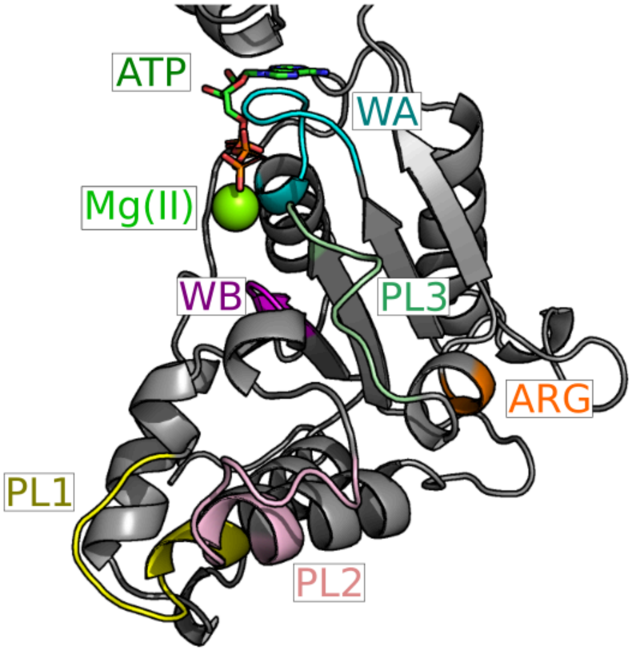

Fig S1. View of katanin NBD detailing the ATP and CTT binding sites.

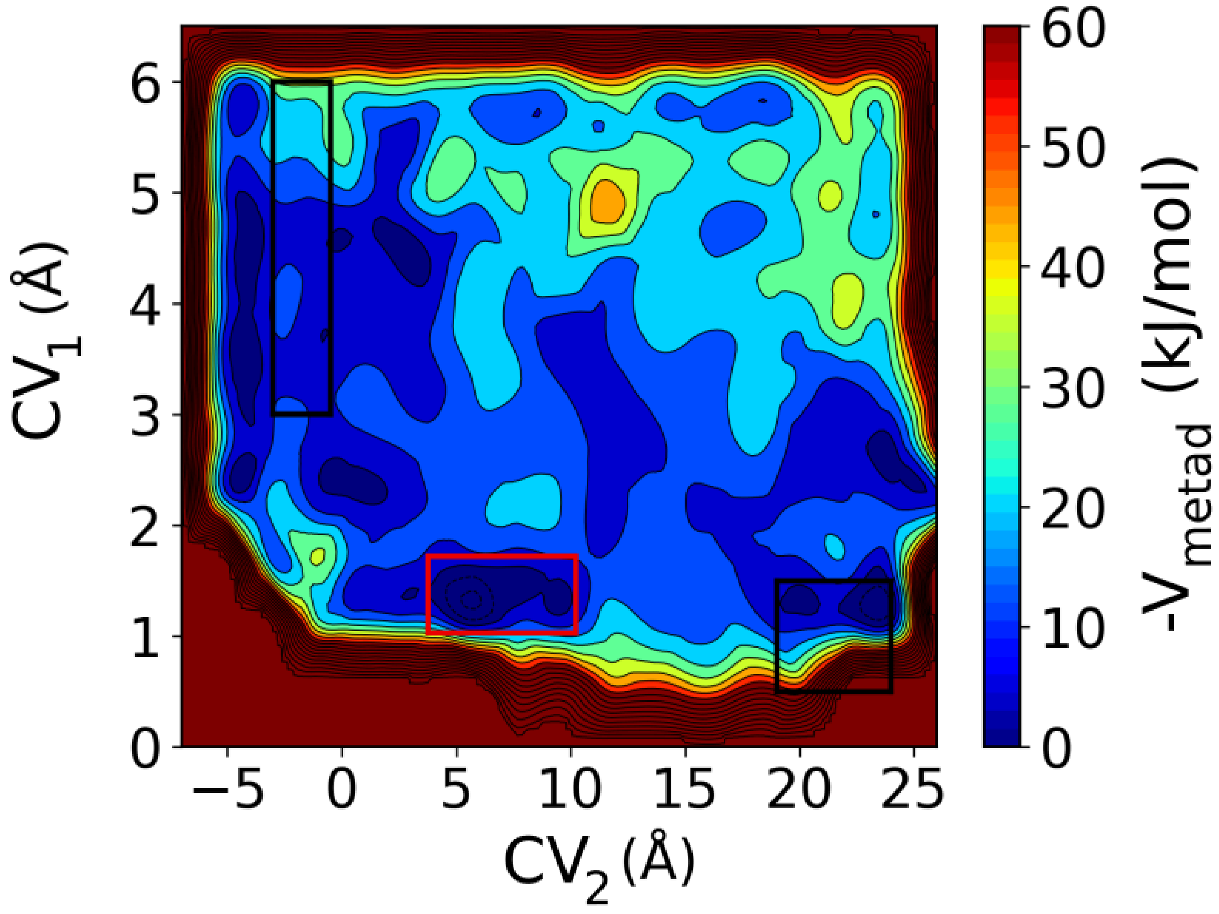

Fig S2. Negative potential deposited during multiple-walker well-tempered Metadynamics simulation. This plot provides a qualitative estimation of the part of the conformational space (projected along the CVs) which are accessible during simulations. The regions in dark blue color between the two stable states (black rectangles) are conformations which are likely to be visited during the S → R transition. The spiral and ring state boxes are defined similarly to Fig. 3 in the main text. The intermediate region is indicated by the red box.

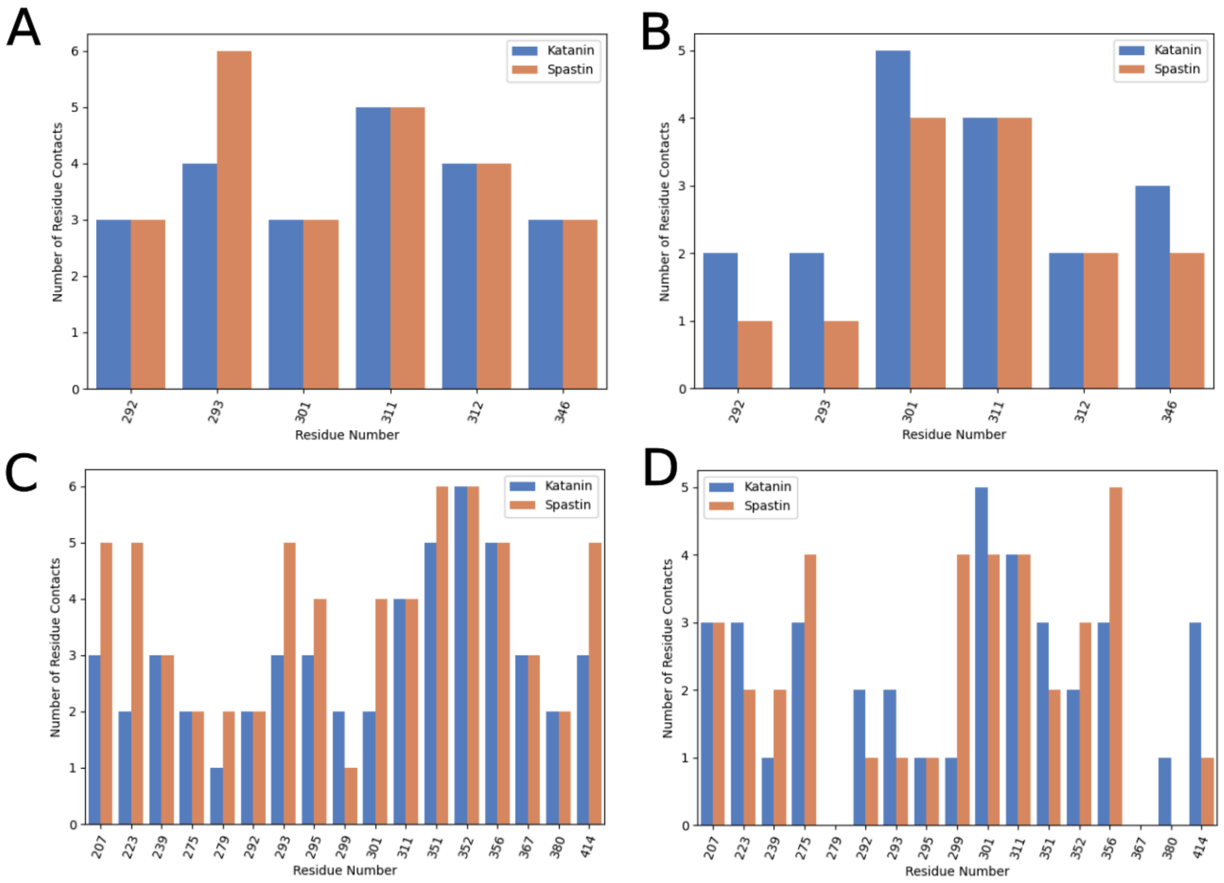

Fig S3. The number of (A,C) **intra** and (B,D) **inter**-protomer contacts calculated for disease-related residues in S-M87 and aligned residues in katanin. Top row shows residues found in CV_2 as important for the S→R, and bottom row shows residues important for the stability of the intermediate. Protomer C of each protein's starting configuration was analyzed to compare the environment of the hexamer where a protomer has neighbors along both interfaces. Katanin residues are used to label the x-axis. S-M87 alignments are listed in Table S1. Contacts for a residue reports the number of unique residues for which at least one pair of heavy atoms, one from the central residue and one from the partner residue, were within 8 Å. The calculations were carried out using the MDAnalysis Python package.[2.]

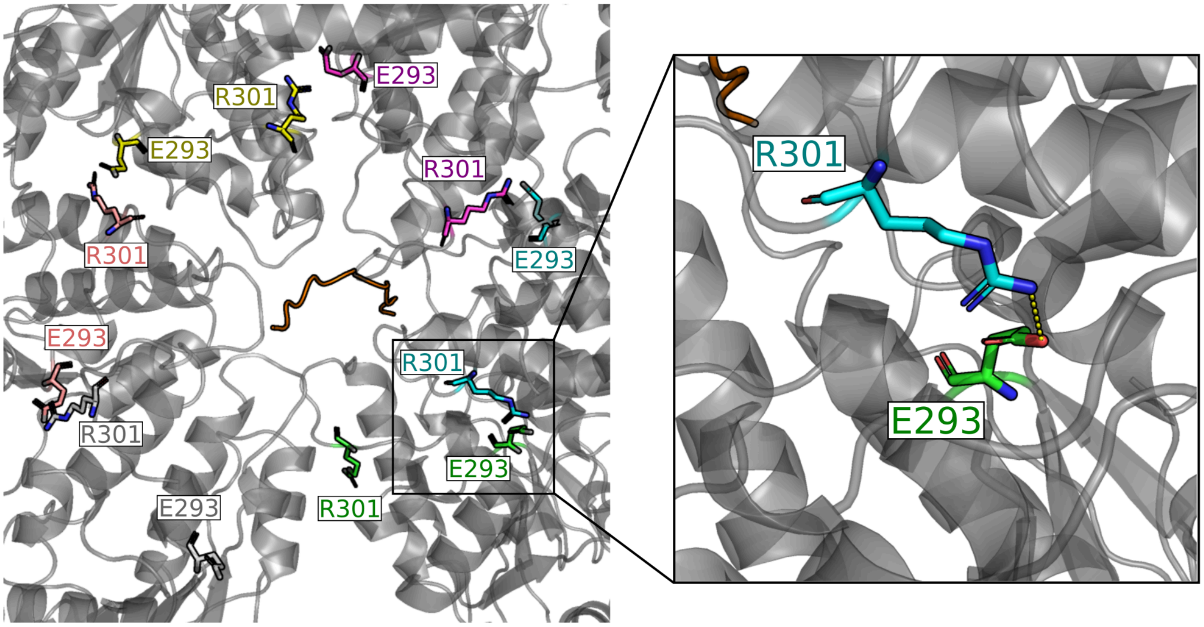

Fig S4. Interactions between R301 and E293 that are in salt bridge distance between protomers.

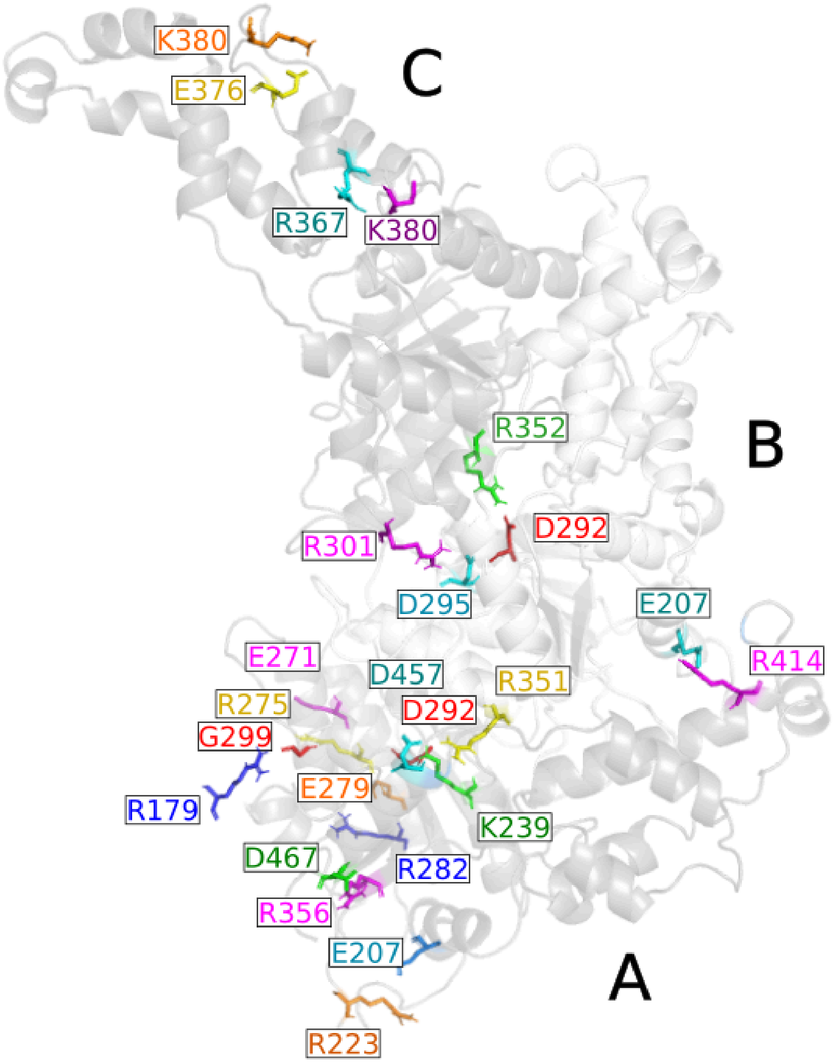

Fig S5. Salt bridges/hydrogen bonds found specifically important for the stability of katanin's intermediate. Protomers A, B, and C shown.

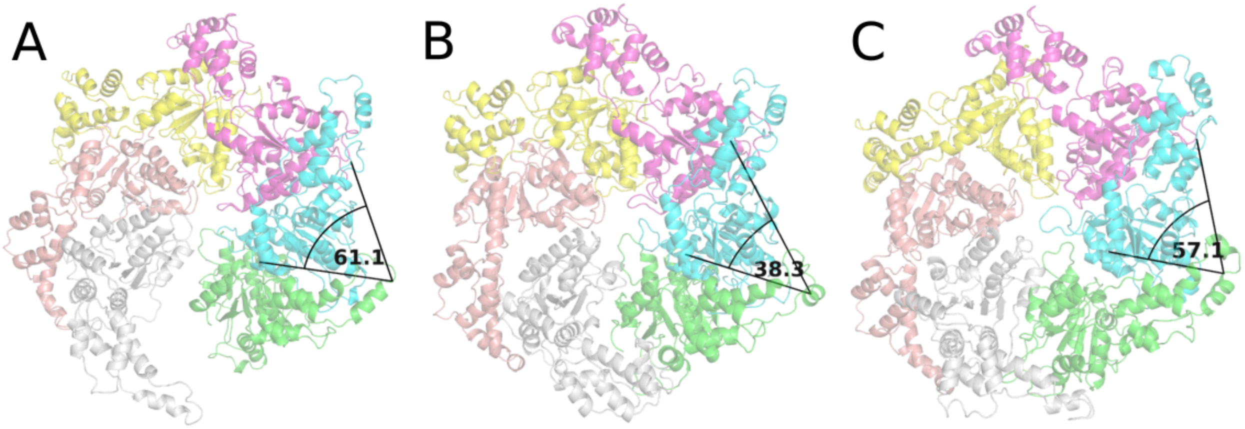

Fig S6. Protomer twist angle of (A) spiral (B) intermediate and (C) ring state.

Tables

S-M87 Residue undergoing mutation(s)	Disease-linked mutations	Impaired Function when Mutated	Corresponding residue in Katanin
**I344**	K	Nucleotide Binding	**I195**
**G346**	C	Nucleotide Binding	**G197**
Q347	K, P	Nucleotide Binding	M198
**E356**	G	Nucleotide Binding	**E207**#
**L360**	P	Oligomerization	**L211**
**P361**	L, S	Oligomerization	**P212**
S362	C	-	R356
R364	M	Oligomerization	V215
L367	S	Oligomerization	F218
**F368**	L, S	Oligomerization	**F219**
T369	P	Oligomerization	Q220
**G370**	R	Oligomerization	**G221**
**R372**	G	Oligomerization	**R223**#
**P374**	R	Oligomerization	**P225**
G377	E	Folding & Stability	A228
L378	G	Folding & Stability	M229
F381	C, L	Folding & Stability	A232
**G385**	E	Nucleotide Binding	**G236**
N386	K	Nucleotide Binding	T237
**K388**	R	Nucleotide Binding	**K239**#
M390	V	Nucleotide Binding	L241
**S399**	L	Folding & Stability	**S250**

S-M87 Residue undergoing mutation(s)	Disease-linked mutations	Impaired Function when Mutated	Corresponding residue in Katanin
I406	V	Folding & Stability	V257
**S407**	R	Oligomerization	**S258**
**G417**	E	Oligomerization	**G268**
**V423**	L	Folding & Stability	**V274**
**R424**	G	Oligomerization	**R275#**
**L426**	V	Folding & Stability	**L277**
**F427**	C	Folding & Stability	**F278**
A428	P	Folding & Stability	E279#
**P435**	T, L	Folding & Stability	**P286**
**S436**	F	Folding & Stability	**S287**
**D441**	G	Mg Coordination	**D292*#**
**E442**	K	Nucleotide Binding	**E293*#**
**D444**	E, N, G	Oligomerization	**D295#**
C448	Y	Oligomerization	G299#
**R450**	S	Oligomerization	**R301*#**
**S458**	R	Folding & Stability	**S310**
**R459**	T	Oligomerization	**R311*#**
**R460**	C, L	Oligomerization	**R312***
L461	P	Folding & Stability	V313
**L466**	V	Folding & Stability	**L318**
**G471**	D	Folding & Stability	**G323**
**N487**	D	Oligomerization	**N340**
**P489**	L	Folding & Stability	**P342**

S-M87 Residue undergoing mutation(s)	Disease-linked mutations	Impaired Function when Mutated	Corresponding residue in Katanin
**D493**	G	Folding & Stability	**D346***
**R498**	S	Phosphate Binding	**R351#**
**F499**	C, H	ATP Hydrolysis	**F352#**
**R503**	L, G	Oligomerization	**R356#**
**R514**	G	Folding & Stability	**R367#**
G527	N	Folding & Stability	K380#
**L537**	P	Folding & Stability	**L389**
**G546**	R	Folding & Stability	**G398**
L549	P	Folding & Stability	V401
A551	Y, P	Oligomerization	S403
D555	N, G	Oligomerization	T407
**A556**	V	Folding & Stability	**A408**
**A557**	V	Folding & Stability	**A409**
G559	D	Oligomerization	N411
R561	G	Oligomerization	L413
**R562**	Q	Oligomerization	**R414#**
N579	H	-	L437
D584	H, V	-	I441
**W607**	C	Folding & Stability	**W465**
D613	H	Oligomerization	A471

Tab S1. Residues of human S-M87 whose mutations (one to three) are associated with hereditary spastic paraplegias. Aligned residues for katanin from C. elegans are listed next to them. The role of each residue determined in experimental mutation studies is listed if known. * indicates that the residue is included in CV_2 (Tab. S2). # indicates that the residue was found

important in the Intermediate along the S→R transition. The residues in boldface are conserved across S-M87 and katanin.[6,8]

Number of interaction	Interaction	Protomers
1#	H307 - E316	A - B
2#	R301 - D261	A - F
3#	E306 - R301	A - B
4#	E271 - R267	A - B
5#	D346 - K265	A - F
6#	D166 - R301	F - A
7#	D171 - R301	F - A
8#	D171 - R312	F - A
9#	D261 - K272	A - B
10#	E347 - K265	B - A
11#	D346 - R301	A - B
12#	E306 - R312	A - B
13#	D346 - R301	F - A
14#	E308 - R312	E - D
15#	D292 - R301	A - B
16#	E306 - R312	B - C
17#	E316 - K265	B - A
18#	D269 - R312	A - A
19#	D457 - R312	C - D
20#	D292 - R312	C - D
21#	E308 - K265	F - F
22#	D171 - K265	B - A

Number of interaction	Interaction	Protomers
23#	D292 - R301	E - F
24#	E293 - R312	A - B
25*	S304 - D171	F - A
26#	D269 - D265	B - A
27#	E308 - R301	C - C
28*	D171 - N303	F - A
29#	E347 - R311	F - F
30#	E306 - R312	D - E
31#	E344 - R301	B - B
32#	D269 - F311	F - A

Tab S2. Intra- or Inter-protomer residue-residue H-bond (*) and salt bridge (#) interactions included in CV_2. The protomers A-F are shown in Fig. 2.

References

1. Macke, Amanda C, Kelly, Maria S, Varikoti, Rohith Anand, Mullen, Sarah, Groves, Daniel, Forbes, Clare, & Dima, Ruxandra I. Microtubule Severing Enzymes Oligomerization and Allostery: A Tale of Two Domains. *The journal of physical chemistry. B*, *126*(50), 10569-10586. (2022).
2. N. Michaud-Agrawal, E. J. Denning, T. B. Woolf, and O. Beckstein. MDAnalysis: A Toolkit for the Analysis of Molecular Dynamics Simulations. *J. Comput. Chem.* 32, 2319-2327. (2011).
3. A Zehr, A Szyk, E Szczesna, A Roll-Mecak, Katanin grips the β-tubulin tail through an electropositive double spiral to sever microtubules. *Dev. Cell* 52, 118–131.e6 (2020).
4. Q Liu, G Zhang, Z Ji, H Lin, Molecular and cellular mechanisms of spastin in neural development and disease (review). Int. J. Mol. Medicine 48 (2021).
5. Rezabkova, K Jiang, G Capitani, , et al., Structural basis of katanin p60:p80 complex formation. Sci. Reports 7, 14893 (2017).

6. CR Sandate, A Szyk, EA Zehr, GC Lander, A Roll-Mecak, An allosteric network in spastin couples multiple activities required for microtubule severing. Nat. Struct. & Mol. Biol. 26,671–678 (2019).
7. S Havlicek, et al., Gene dosage-dependent rescue of HSP neurite defects in SPG4 patients' neurons. Hum. Mol. Genet. 23, 2527–2541 (2013).
8. Han H, Schubert HL, McCullough J, Monroe N, Purdy MD, Yeager M, Sundquist WI, Hill CP. Structure of spastin bound to a glutamate-rich peptide implies a hand-over-hand mechanism of substrate translocation. J Biol Chem. Jan 10;295(2):435-443. (2020).
